# Atypical MRI presentation of symmetrical thalamic and basal ganglia lesions in adult-onset MOG-IgG-associated disease: a case report

**DOI:** 10.3389/fnhum.2026.1897425

**Published:** 2026-08-19

**Authors:** Yutan Wang, Zikai Xin, Ruijie Meng, Zhihong Zhao, Zilong Zhu

**Affiliations:** 1 Clinical College of Neurology, Neurosurgery, and Neurorehabilitation, Tianjin Medical University, Tianjin, China; 2 Tianjin Key Laboratory of Cerebral Vascular and Neurodegenerative Diseases, Huanhu Hospital, Tianjin University, Tianjin, China

**Keywords:** adult-onset, case report, demyelinated lesions, MOGAD, MRI presentation

## Abstract

Myelin oligodendrocyte glycoprotein antibody-associated disease (MOGAD) is a rare autoimmune neurological condition characterized by clinical phenotypes that include acute disseminated encephalomyelitis, optic neuritis, and transverse myelitis. Neuroimaging features differ markedly between adults and children, and brain lesions are relatively uncommon in adult MOGAD patients. Atypical imaging patterns can complicate the differentiation of MOGAD from other diseases. We report a 21-year-old female presenting with dysarthria, gait deviation, blurred vision, and slow response. Brain MRI revealed multiple patchy, irregular T2-FLAIR hyperintense lesions involving the pons, midbrain, and strikingly symmetric bilateral thalami and basal ganglia. She had a history of chronic alcohol use and was initially misdiagnosed with Wernicke’s encephalopathy. No symptomatic improvement followed vitamin B1 supplementation. Serum and cerebrospinal fluid (CSF) analysis revealed low-positive myelin oligodendrocyte glycoprotein immunoglobulin G (MOG-IgG). After carefully excluding other differential diagnoses, a diagnosis of MOGAD was established. High-dose intravenous methylprednisolone followed by oral tapering resulted in significant clinical improvement, along with marked amelioration of MRI abnormalities. This case highlights an atypical adult MOGAD presentation with widespread brainstem to the basal ganglia and symmetric thalamic lesions. It underscores the importance of recognizing such imaging patterns to avoid misdiagnosis and prompt timely autoantibody testing and immunotherapy.

## 1 Introduction

Myelin oligodendrocyte glycoprotein antibody-associated disease (MOGAD) is an autoimmune demyelinating disorder of the central nervous system (CNS) that presents with a wide spectrum of clinical phenotypes, including optic neuritis (ON), transverse myelitis, acute disseminated encephalomyelitis (ADEM), brainstem encephalitis, and cortical encephalitis (Zhang and Li, 2025). Although MOGAD can affect both adults and children, its clinical and radiological features differ significantly between the two groups. In adults, isolated ON is the most common presentation, whereas ADEM-like manifestations occur more frequent in children (Li et al., 2023; Lei et al., 2022; Chen et al., 2018; Wang et al., 2022; Jurynczyk et al., 2017). Brain lesions are relatively uncommon in adult MOGAD patients, and when present, they typically involve the brainstem, cerebellar peduncles, or periventricular regions, often with a “fluffy” appearance (Lei et al., 2022; Jurynczyk et al., 2017).

Despite increasing recognition of MOGAD, its imaging findings remain highly heterogeneous, and no single radiological feature is pathognomonic for the disease (Sechi et al., 2022). This poses a diagnostic challenge, particularly in atypical cases, and may lead to misdiagnosis or delayed treatment. However, if cell-based MOG-IgG testing methods are widely used without appropriate clinical or radiological filtering can result in false-positive results, especially at low titers (Sechi et al., 2022; Sechi et al., 2021; Cortese et al., 2023a). Therefore, identifying imaging patterns that warrant autoantibody testing is of great clinical importance.

Herein, we report an unusual case of adult-onset MOGAD in a 21-year-old female, characterized by widespread involvement from the brainstem to the basal ganglia and strikingly symmetric lesions in the thalami and basal ganglia on brain MRI This pattern is rarely seen in adult patients and therefore easily mistaken for other disorders. This case underscores the importance of recognizing such atypical features to avoid misdiagnosis and to facilitate the timely initiation of autoantibody testing and immunotherapy.

## 2 Case presentation

A 21-year-old female, working as an alcohol salesperson with a long history of alcohol use, presented with a six-day history of dysarthria and communication difficulties, though language comprehension remained intact. Three days prior to admission, she developed slowed response, blurred vision in both eyes, and unsteady gait. She also reported a febrile episode (maximum temperature 39 °C) 2 months before admission, which resolved after 1 week of symptomatic treatment. Neurological physical examination revealed sluggish responses, dysarthria, horizontal nystagmus on rightward gaze, left-sided limb weakness, a positive left Babkin sign, and ataxia. She underwent cognitive examination, which indicated mildly impaired cognitive function, as evidenced by a Mini-Mental State Examination (MMSE) score of 24/30.

**Figure 1:**
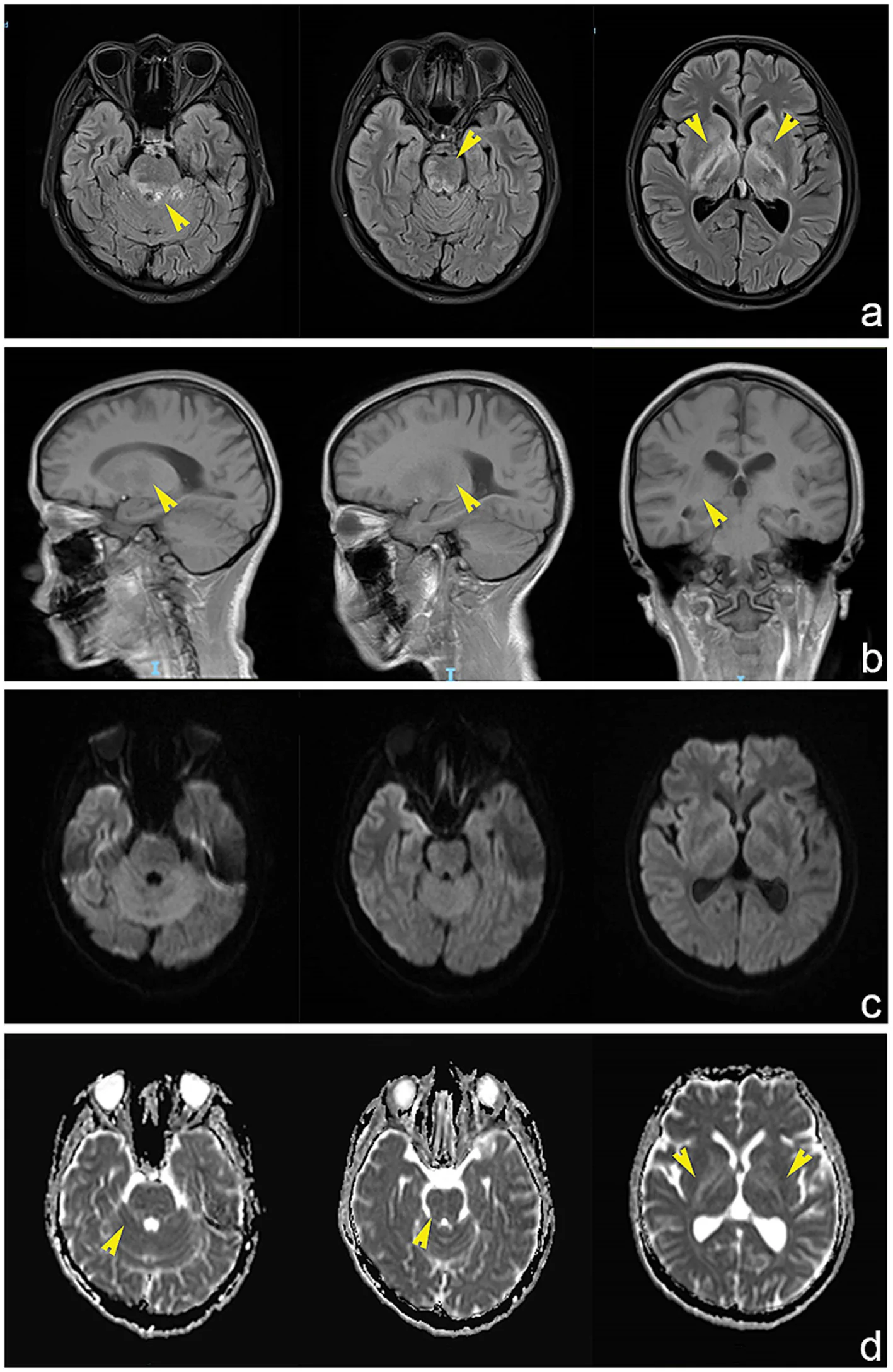
Brain MRI of the patient. **(a)** FLAIR sequences revealed multiple hyperintense lesions (yellow arrows) involving the pons, bilateral middle cerebellar peduncles, bilateral thalami, and bilateral basal ganglia. The lesions were hypointense on T1WI **(b)** and appeared isointense on DWI images **(c)** and hyperintense on ADC maps **(d)**.

**Figure 2:**
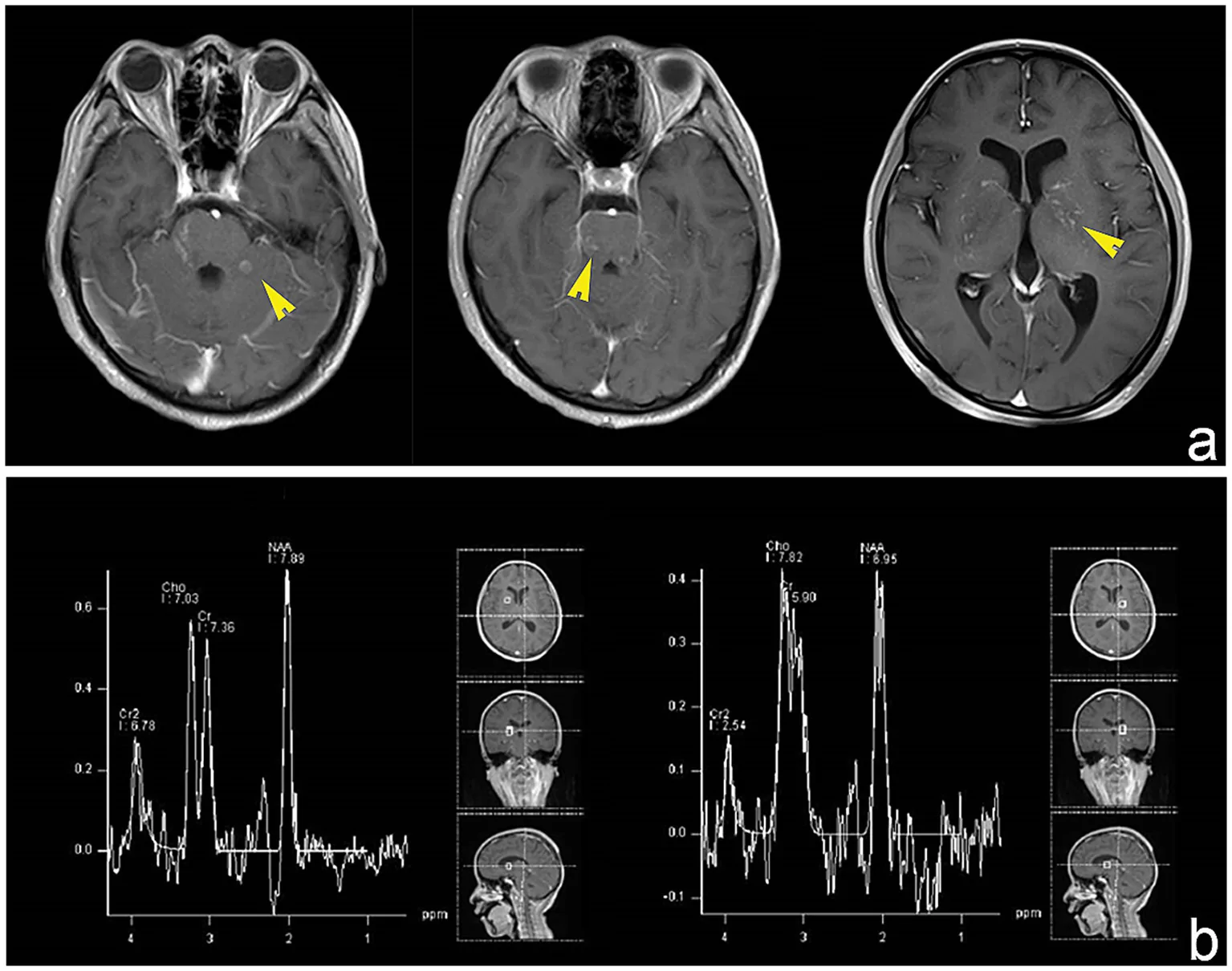
Contrast-enhanced brain MRI and MRS of the patient **(a)** Contrast-enhanced brain MRI showed multiple punctate and irregular abnormal enhancing lesions (yellow arrows). **(b)** MRS indicated a significant decrease in N-acetylaspartate (NAA)/creatine (Cr) ratio and an increased choline (Cho)/Cr ratio in the lesion area.

Brain magnetic resonance imaging (MRI) revealed multiple patchy, irregular T2-fluid-attenuated inversion recovery (FLAIR) hyperintense lesions involving the pons, bilateral middle cerebellar peduncles, bilateral thalami, and bilateral basal ganglia, with strikingly symmetric involvement of the thalami and basal ganglia. These lesions were hypointense on T1-weighted imaging (T1WI) and appeared isointense on diffusion-weighted imaging (DWI), and hyperintense on apparent diffusion coefficient (ADC) maps. The margins of these lesions were slightly blurred, with no mass effect or significant surrounding edema (Figure 1). Contrast-enhanced brain MRI showed punctate and irregular abnormal enhancement signals in the lesion area (Figure 2a). A multi-voxel MRS was performed using a point-resolved spectroscopy (PRESS) sequence with TR of 1700 ms, TE of 135 ms, and FOV of 16 cm × 16 cm. A single cubic voxel of 1.0 × 1.0 × 1.5 cm (1.5 cm^3^) was carefully positioned centrally within the contrast-enhancing lesion involving the bilateral basal ganglia, avoiding contamination from adjacent cerebrospinal fluid spaces and normal-appearing white matter. The MRS revealed a significant decrease in the N-acetylaspartate (NAA)/creatine (Cr) ratio and an increased choline (Cho)/Cr ratio within the targeted lesion (Figure 2b). These metabolic alterations indicate neuronal/axonal injury and active membrane turnover. Magnetic resonance venography (MRV) revealed no significant stenosis, interruption, filling defects, or other vascular abnormalities within the intracranial deep venous system (data not shown). All baseline laboratory investigations—including routine hematology, coagulation, thyroid and pituitary function, autoantibodies (ANA, ANCA), infectious serology (syphilis, HIV), and tumor markers (CEA, AFP, CA19-9, CA72-4, CK19)—yielded normal results. Serum interleukin-6 was mildly elevated (IL-6, 13.99 pg./mL). We also performed chest CT and abdominal and thyroid ultrasound examinations on this patient, which revealed no abnormalities. In addition, a visual assessment revealed vision loss in both eyes--the visual acuity was 20/40 in the left eye and 20/30 in the right eye. Visual field examination revealed a few irregular scotomas in the right eye, a partial superior visual field defect in the left eye, and scattered scotomas in the inferior visual field. Visual evoked potential (VEP) showed prolonged latency of the P100 wave, and optical coherence tomography (OCT) revealed thinning of the retinal nerve fiber layer (RNFL) in the left eye (data not shown).

Given the patient’s history of chronic alcohol use, normal body temperature, absence of toxin or heavy metal exposure, no history of specific medication use, and no complaints of exercise intolerance, combined with the laboratory and MRI findings, Wernicke’s encephalopathy was initially considered. However, vitamin B1 supplementation produced no symptomatic improvement, and subsequent blood tests for B vitamins were normal. Lumbar puncture and cerebrospinal fluid (CSF) analysis were performed to further characterize the diagnosis and clarify the underlying etiology on hospital day 5. The results revealed normal intracranial pressure (100 mmH₂O) and normal white cell count (8 × 10^6^/L), glucose (2.52 mmol/L), and chloride (123 mmol/L), but protein levels were mildly increased (0.47 g/L). Pathogen testing (including acid-fast staining, fungal and bacterial smears, and India ink staining) was negative. CSF IgG index mildly elevated (0.88). Oligoclonal bands were detected in CSF but not in serum.

**Figure 3:**
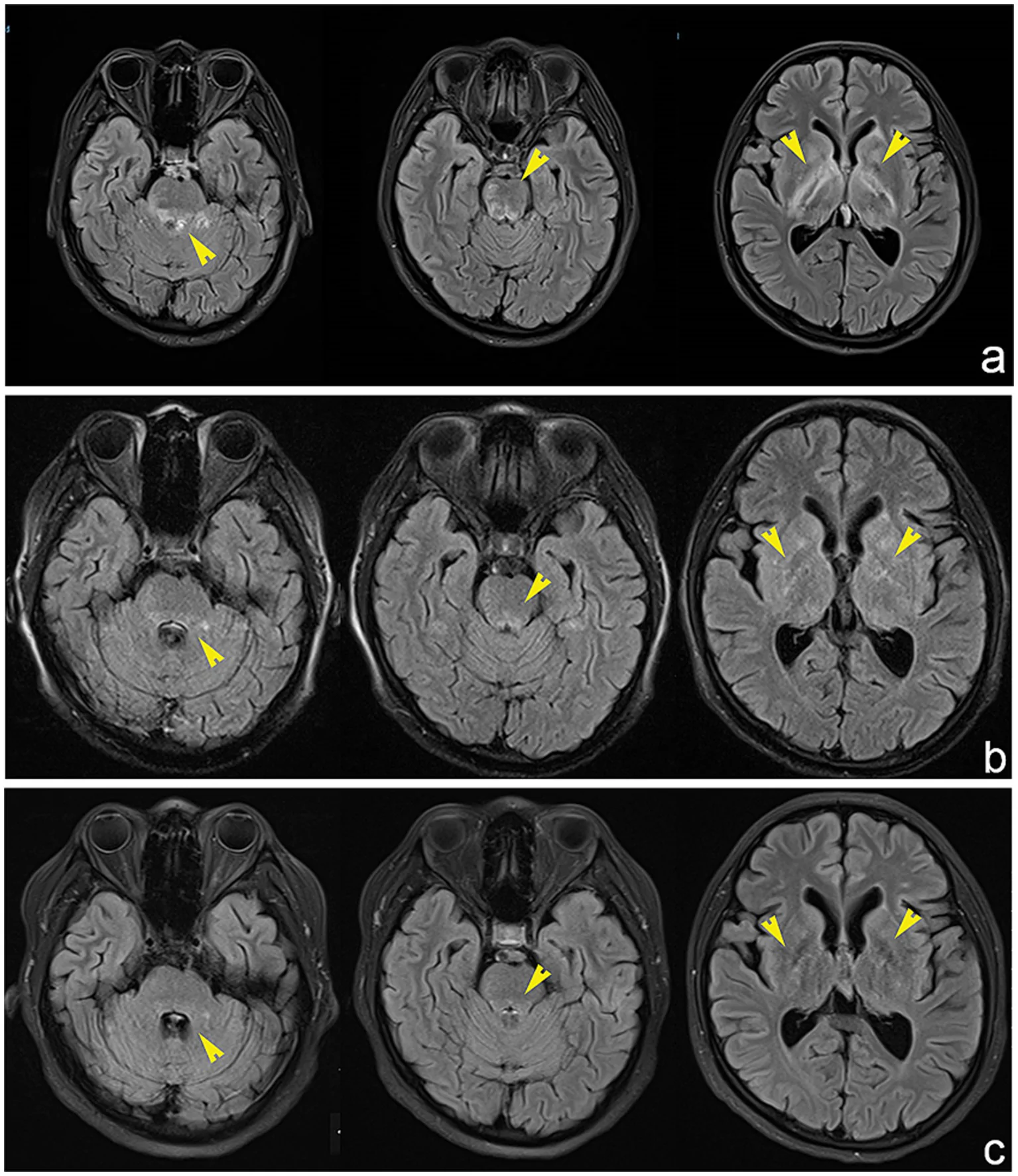
MRI showed marked reduction or complete resolution of the abnormal signals at one- and three-month post-treatment. **(a)** Before treatment, FLAIR images revealed multiple hyperintense lesions (yellow arrows) involving the pons, bilateral middle cerebellar peduncles, thalami, and basal ganglia. **(b)** At 1 month post-treatment, the lesions (yellow arrows) had dramatically shrunk. **(c)** At 3 months post-treatment, FLAIR sequences revealed that the original abnormal signals (yellow arrows) were largely obliterated.

**Figure 4:**
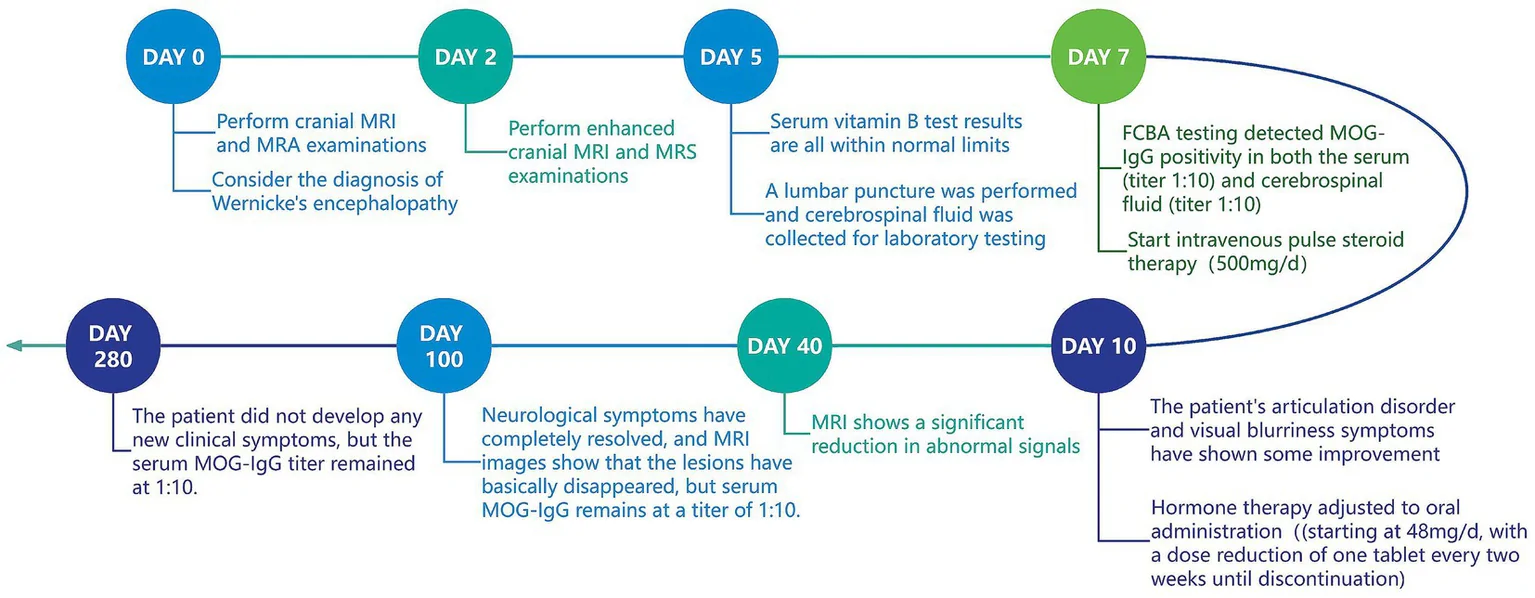
The timeline of this patient’s laboratory findings, MRI examinations, and treatment.

Autoantibody testing revealed the presence of myelin oligodendrocyte glycoprotein immunoglobulin G (MOG-IgG) positivity in both serum (titer 1:10) and cerebrospinal fluid (titer 1:10), while other autoimmune encephalitis antibodies, including aquaporin-4 immunoglobulin G (AQP4-IgG), were all negative. MOG-IgG was detected using a fixed cell-based assay with HEK293 cells expressing full-length human MOG. Serum and CSF samples were screened at a 1:10 dilution. Bound MOG-IgG was visualized using a fluorescein isothiocyanate (FITC)-conjugated anti-human IgG secondary antibody. The laboratory cutoff for positivity was ≥1:10, and our patient’s titer of 1:10 in both serum and CSF was reported as positive (low-positive) according to the laboratory’s established criteria. According to the 2023 diagnostic criteria proposed by the International MOGAD Panel (Banwell et al., 2023), the patient presented with a core clinical demyelinating event (brainstem- and cerebellum-related clinical neurological deficits), the cell-based serum assay for MOG-IgG yielded a low-positive result (titer ≥1:10 and <1:100 on fixed cell assay) (Budhram and Flanagan, 2025; Redenbaugh et al., 2024), and AQP4-IgG testing was negative, additionally, supportive magnetic resonance imaging (MRI) features were observed, including involvement of the deep gray matter and ill-defined T2 hyperintensities in the pons and middle cerebellar peduncles. After excluding other differential diagnoses, a diagnosis of myelin oligodendrocyte glycoprotein antibody-associated disease (MOGAD) was established. Subsequently, the patient received high-dose intravenous methylprednisolone (500 mg/day for three consecutive days). By the third day of treatment, both dysarthria and blurred vision had noticeably improved relative to baseline, no significant adverse reactions were observed. Then, treatment was switched to a tapering oral regimen of methylprednisolone, starting at 48 mg daily and reduced by one tablet every 2 weeks until discontinuation. At one-month follow-up, MRI revealed that the abnormal signals had dramatically shrunk (Figures 3a,b). At three-month follow-up, her neurological symptoms had achieved complete resolution, and MRI imaging showed the lesion was largely obliterated (Figures 3a,c), however, the serum MOG-IgG titer remained at 1:10. At the nine-month follow-up, the patient exhibited no new clinical symptoms, although the serum MOG-IgG titer persisted at 1:10. The timeline of this patient’s laboratory findings, MRI examinations, and treatment is shown in Figure 4.

## 3 Discussion

We report an unusual case of adult-onset MOGAD in a 21-year-old female presenting with widespread brainstem-to-basal ganglia involvement and strikingly symmetric thalamic and basal ganglia lesions on brain MRI. This case illustrates the phenotypic variability of MOGAD and highlights the diagnostic difficulties posed by atypical neuroimaging features in adult patients.

MOGAD can occur at any age, but its clinical features differ markedly between adult and pediatric patients (Li et al., 2023; Lei et al., 2022; Chen et al., 2018; Wang et al., 2022; Jurynczyk et al., 2017). In adult patients, the clinical manifestations are predominantly ON and myelitis, with ON being the most common initial symptom (Li et al., 2023; Chen et al., 2018). Brain lesions are relatively uncommon in adult patients. In a cohort study by Jurynczyk et al., only 7% of adult MOGAD patients had brainstem-to-basal ganglia involvement, and 74% had three or fewer intracranial lesions (Jurynczyk et al., 2017). Similarly, Cortese et al. (2023b) reported that the likelihood of MOGAD decreases with increasing lesion number in adult patients. In pediatric patients, however, the clinical presentation is predominantly ADEM. A Chinese study reported that approximately 68.8% of pediatric MOGAD patients had brain lesions (Chen et al., 2018). Another study showed that among 48 pediatric patients with MOGAD, 20 (41.7%) presented with ADEM-like lesions (Wang et al., 2022). In addition, pediatric patients with MOGAD are more likely to exhibit bilateral, large, brainstem-to-basal lesions (Jurynczyk et al., 2017). These findings were further corroborated by Trewin and colleagues in a large global cohort (Trewin et al., 2025). The mechanisms underlying the differences in MOGAD manifestations between adults and children may involve multiple factors, including immune system maturity, MOG antibody characteristics, and CNS developmental status. First, children are in a critical period of immune system development and maturation, and their immune response patterns differ markedly from those of adults. A study by Ohashi et al. found that pediatric MOGAD patients exhibit higher Th17 cell activity in the cerebrospinal fluid (CSF), as reflected by elevated levels of Th17-related cytokines, including interleukin-6 and chemokine ligand 8, which correlate with encephalitic phenotypes such as ADEM (Ohashi et al., 2026). Additionally, pediatric patients with MOGAD also have a higher incidence of eosinophils in the CSF. One study reported that approximately one-third of affected children exhibit eosinophils in the CSF, suggesting that the immunopathological mechanisms of pediatric MOGAD may involve a broader range of immune cell types (Kornbluh et al., 2024). Furthermore, concerning CNS developmental status, the CNS of children is in the myelination stage, during which MOG expression levels are higher, making it a more vulnerable target for antibody attack (Bruijstens et al., 2020). Notably, the neuroimaging characteristics of our case are the combination of widespread supratentorial and infratentorial lesions, including brainstem, bilateral cerebellar peduncles, midbrain, bilateral thalami, and basal ganglia, with a total of eight lesions, which exceeds the typical radiographic feature of three or fewer lesions often associated with adult MOGAD in previous studies. In contrast, our patient’s lesion count and distribution more closely resemble those of pediatric MOGAD. This was one of the factors contributing to our early misdiagnosis. We analyzed the possible reasons, one of which was that our patient’s family history of slower developmental milestones (late walking and talking) and persistent mild cognitive difficulties, which prompted us to propose the hypothesis that neurodevelopmental delay in our patient might reflect a more “immature” pattern of brain injury in MOGAD. Nevertheless, this interpretation is constrained by the absence of objective developmental assessments. Moreover, it is highly speculative, being based on a single case, and requires validation in larger, well-phenotyped cohorts. In addition, previous studies and predictive models have typically employed a simple adult/child dichotomy for stratification, which may reduce diagnostic accuracy. Therefore, future studies should incorporate more refined age subgroups to better understand the clinic-radiological heterogeneity of MOGAD, thereby facilitating timely autoantibody testing and immunotherapy and avoiding misdiagnosis.

Another important aspect of this case is the remarkable symmetry of the thalamic and basal ganglia lesions. Symmetric deep gray matter involvement can be observed in various conditions, including Wernicke encephalopathy, toxic-metabolic encephalopathy, acute disseminated encephalomyelitis (ADEM), aquaporin-4 IgG-positive neuromyelitis optica spectrum disorder (AQP4-IgG^+^ NMOSD), deep venous thrombosis, and mitochondrial diseases. Our patient had a long history of alcohol use, which initially led to a misdiagnosis of Wernicke’s encephalopathy. However, the lack of response to thiamine supplementation and normal vitamin B1 levels do not support this diagnosis. Furthermore, although visual impairment is also relatively common in alcohol abuse (e.g., significant prolongation of P100 wave latency and reduced amplitude on VEP, as well as significant thinning of the peripapillary RNFL thickness), visual impairment in patients with chronic alcohol abuse predominantly involves central visual acuity loss, and visual field defects typically present as central or paracentral scotomas (Delibes et al., 2024; Xie et al., 2022; Nicolaou et al., 2025). This difference can serve as one of the distinguishing features between the two conditions. In addition, given the patient’s normal body temperature on admission, and the absence of any specific medical history (such as exercise intolerance, toxic or drug exposure, or factors predisposing to a hypercoagulable state), the aforementioned differential diagnoses were progressively excluded based on imaging and laboratory findings. Involvement of the middle cerebellar peduncles is also associated with multiple conditions, such as multiple sclerosis (MS), AQP4-IgG^+^ NMOSD, multiple system atrophy (MSA), and spinocerebellar ataxia (SCA) (Shahriari et al., 2021; Tamanini et al., 2024). In this case, the MRI findings consisted of multiple patchy and irregular T2-FLAIR hyperintense lesions with slightly ill-defined margins, no mass effect, no significant perilesional edema, and no obvious cerebral atrophy, along with partial gadolinium enhancement. Moreover, although the MRS findings—showing a decreased NAA/Cr ratio and an elevated Cho/Cr ratio—lacked diagnostic specificity (not diagnostic marker for MOGAD), these spectroscopic data were interpreted as evidence of an acute, metabolically active parenchymal process—a finding more commonly associated with inflammatory, demyelinating, neoplastic, or other destructive conditions (Takenaka et al., 2011; Aboul-Enein et al., 2010). Taken together with the positive serum MOG-IgG antibody test, other relevant laboratory results, and the marked clinical response to corticosteroid therapy, the diagnosis of MOGAD was ultimately established after the exclusion of the above differential diagnoses.

It is worth noting that the patient’s serum MOG-IgG titer remained low-positive (1:10) during follow-up, despite marked clinical and radiographic improvement. This finding aligns with previous reports suggesting that MOG antibody titers do not necessarily correlate with disease activity or treatment response (Banwell et al., 2023; Cobo-Calvo et al., 2019; Akaishi et al., 2019). Therefore, clinical and radiological assessments should remain the primary basis for therapeutic decisions, and persistent low-level seropositivity should not be overinterpreted as evidence of ongoing active disease. Several limitations should be acknowledged. First, the patient refused spinal cord MRI due to the absence of spinal cord-related symptoms and financial constraints, which limited our ability to assess subclinical spinal cord lesions. Second, the absence of a reliable visual history and more detailed neuro-ophthalmological data, including visual acuity using a standard notation, color vision, fundoscopic examination, relative afferent pupillary defect, optic nerve/orbital MRI findings with optic nerve enhancement status, constrains our assessment of the optic neuropathy, a key component in establishing both the diagnosis and differential diagnosis of MOGAD. Third, a longer follow-up is needed to monitor for relapses and to confirm the durability of radiographic improvement. Lastly, as a single case report, the hypotheses generated require validation in larger, well-phenotyped cohorts. Despite these limitations, our case provides valuable insights into the atypical imaging spectrum of adult MOGAD and underscores the importance of recognizing symmetrical deep gray matter and extensive brainstem involvement as potential red flags for this condition.

## 4 Conclusion

In summary, we report an adult MOGAD patient with extensive, symmetric deep gray matter and brainstem lesions that more closely resemble the pediatric imaging phenotype. This case expands the known radiological spectrum of adult MOGAD and highlights the diagnostic challenge posed by atypical features, particularly when clinical history includes potential confounders such as alcohol use. Future studies may stratify patients by detailed age subgroups and consider neurodevelopmental factors to better understand the radiological heterogeneity of MOGAD, thereby facilitating timely autoantibody testing to avoid misdiagnosis.

## Data Availability

The original contributions presented in the study are included in the article/Supplementary material, further inquiries can be directed to the corresponding author.
